# Characterizing the Interaction between the HTLV-1 Transactivator Tax-1 with Transcription Elongation Factor ELL2 and Its Impact on Viral Transactivation

**DOI:** 10.3390/ijms222413597

**Published:** 2021-12-18

**Authors:** Stephan Kohrt, Sarah Strobel, Melanie C. Mann, Heinrich Sticht, Bernhard Fleckenstein, Andrea K. Thoma-Kress

**Affiliations:** 1 FAU Junior Research Group “Retroviral Pathogenesis” and BMBF Junior Research Group in Infection Research “Milk Transmission of Viruses”, Institute of Clinical and Molecular Virology, Friedrich-Alexander-Universität Erlangen-Nürnberg (FAU), 91054 Erlangen, Germany; stephan.kohrt@fau.de; 2 Institute of Clinical and Molecular Virology, Friedrich-Alexander-Universität Erlangen-Nürnberg (FAU), 91054 Erlangen, Germany; sarah.strobel@fau.de (S.S.); melanie-mann@gmx.net (M.C.M.); 3 Division of Bioinformatics, Institute of Biochemistry, Friedrich-Alexander-Universität Erlangen-Nürnberg (FAU), 91054 Erlangen, Germany; heinrich.sticht@fau.de

**Keywords:** human T-cell leukemia virus type 1, HTLV-1, ELL2, transcription elongation factor for RNA polymerase II, Tax-1, Tax-2, viral transactivation, viral oncoprotein

## Abstract

The human T-cell leukemia virus type 1 (HTLV-1)-encoded transactivator and oncoprotein Tax-1 is essential for HTLV-1 replication. We recently found that Tax-1 interacts with transcription elongation factor for RNA polymerase II 2, ELL2, which enhances Tax-1-mediated transactivation of the *HTLV-1* promotor. Here, we characterize the Tax-1:ELL2 interaction and its impact on viral transactivation by confocal imaging, co-immunoprecipitation, and luciferase assays. We found that Tax-1 and ELL2 not only co-precipitate, but also co-localize in dot-like structures in the nucleus. Tax-1:ELL2 complex formation occurred independently of Tax-1 point mutations, which are crucial for post translational modifications (PTMs) of Tax-1, suggesting that these PTMs are irrelevant for Tax-1:ELL2 interaction. In contrast, Tax-1 deletion mutants lacking either N-terminal (aa 1–37) or C-terminal regions (aa 150–353) of Tax-1 were impaired in interacting with ELL2. Contrary to Tax-1, the related, non-oncogenic Tax-2B from HTLV-2B did not interact with ELL2. Finally, we found that ELL2-R1 (aa 1–353), which carries an RNA polymerase II binding domain, and ELL2-R3 (aa 515–640) are sufficient to interact with Tax-1; however, only ELL2-truncations expressing R1 could enhance Tax-1-mediated transactivation of the HTLV-1 promoter. Together, this study identifies domains in Tax-1 and ELL2 being required for Tax-1:ELL2 complex formation and for viral transactivation.

## 1. Introduction

Human T-cell leukemia virus type 1 (HTLV-1) is a highly oncogenic delta-retrovirus predominantly infecting CD4^+^ T cells and causing an aggressive type of cancer called adult T-cell leukemia/lymphoma (ATL) or an inflammatory neurological disease leading to damage of the spinal cord, known as HTLV-1-associated myelopathy/tropical spastic paraparesis (HAM/TSP) [1,2,3,4]. The virus is endemic in Japan, sub-Saharan Africa, South America, the Caribbean, parts of the Middle East, Melanesia, and Central Australia, and at least 5–10 million people are infected worldwide [5]. Due to very inefficient cell-free transmission, the virus is mainly transmitted via cell–cell contacts at the virological synapse, via viral biofilms or via cellular protrusions [6,7,8]. The transmission from one person to another can occur sexually, via cell-containing blood products or from mother to child, predominantly via breast-feeding; thus, HTLV-1 belongs to the group of blood-borne viruses and sexually transmissible infections [9,10]. Upon infection, HTLV-1 integrates in vivo into the host cell genome in its provirus form (ca. 9.1 kb), which is flanked by two long terminal repeats (LTRs), 5′ LTR and 3′ LTR [11]. Interestingly, HTLV-1 can persist over decades in the host without causing symptoms. HTLV-1 infection is not part of sexual health screening in most countries; thus, asymptomatic carriers are mainly unaware of their infection and may pass the virus to other people [12]. During latency, HTLV-1 shows reduced gene expression without expression of the immunodominant viral Tax protein, allowing the virus to escape from the CD8^+^ cytotoxic T-cell response (CTL) of the host, which is directed towards the immunogenic Tax-1 protein [13,14,15,16].

The viral oncoprotein Tax-1, encoded by the pX region of HTLV-1, functions not only as a potent transactivator of viral gene expression [17] but also as a viral oncoprotein and initiator of the malignant transformation of infected CD4^+^ T cells. Briefly, Tax-1 fosters aberrant growth of infected cells, interferes with cell cycle control and apoptosis regulation, and induces DNA damage by interacting with a plethora of host cell proteins [18,19,20]. As an essential factor for stimulating viral replication, Tax-1 recruits host cell factors such as positive transcription elongation factor b (p-TEFb) to the viral long terminal repeat (LTR) promoter [21]. The multiprotein complex p-TEFb is a cyclin-dependent kinase consisting of CDK9 as the catalytic subunit and either cyclin T1 or T2 as the regulatory subunit [22,23]. In contrast to incomplete, small and short-lived transcripts generated by RNA polymerase II (RNA Pol II) under the control of negative transcription elongation factors (N-TEFs), p-TEFb catalyzes the transition into productive elongation by the phosphorylation of the carboxyterminal domain of RNA Pol II, thus allowing the production of full-length mRNA transcripts [14,24].

The control of transcription elongation is central to gene regulation because deregulation of this highly orchestrated transcription elongation process often promotes cancer pathogenesis [25]. The transcription elongation factor 2 (ELL2), a member of the eleven-nineteen-rich leukemia family of elongation factors (ELL family), is important for productive transcription. ELL family members increase the catalytic rate and suppress the transient pausing of RNA polymerase II by keeping the 3′OH end of the nascent mRNA on the right track in the center of the RNA Pol II [26,27,28]. The short-lived protein ELL2 can be stabilized by the scaffold protein AFF4 and it is the stoichiometrically limiting factor of a so-called super elongation complex (SEC) that had been identified in the context of HIV transactivation [27,29]. Further, ELL2 is involved in splicing of thousands of genes in B cells and directly influences the immunoglobulin (Ig) heavy chain genes to change the pattern of RNA processing from the membrane specific form in B cells to the secretory-specific mRNA in antibody secreting cells [30,31,32]. We showed that ELL2 is upregulated in HTLV-1-infected cells, interacts with Tax-1 and enhances the Tax-1-mediated transactivation of the HTLV-1-promotor [33]. However, it is still unknown which domains of Tax-1 and ELL2 are necessary for Tax-1:ELL2 complex formation.

Here, we characterize Tax-1:ELL2 complex formation and find that ELL2 does not complex with the related Tax-2 from the non-oncogenic HTLV-2. We identify domains in Tax-1 and ELL2 that are crucial for the interaction and for ELL2’s capacity to foster Tax-1-mediated viral transactivation. Especially ELL2-R1, which carries an RNA polymerase II binding domain, is sufficient to interact with Tax-1 and required for full viral transactivation.

## 2. Results

### 2.1. Tax-1 and ELL2 Co-Localize in Dot-like Structures in the Nucleus

**Figure 1:**
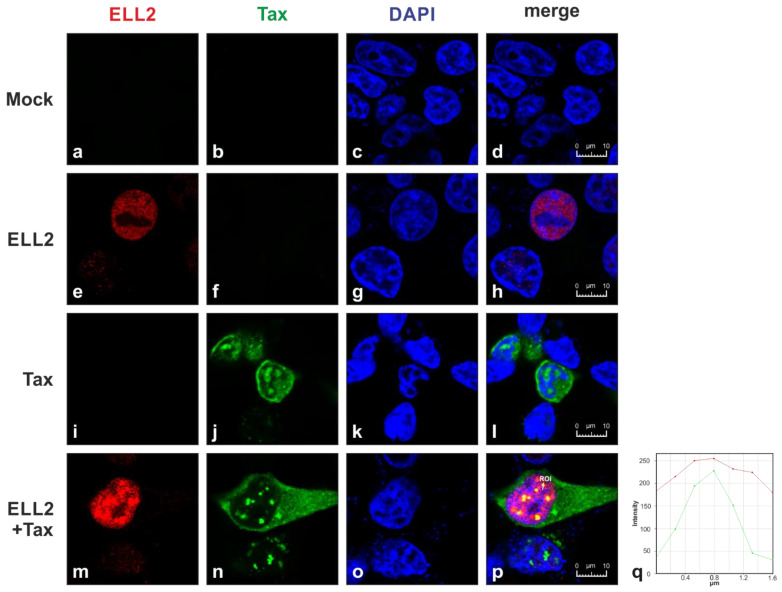
**Tax-1 and ELL2 co-localize in dot-like structures in the nucleus**. (**a**–**p**) Indirect immunofluorescence analysis of ELL2, Tax-1 and the nucleus was performed. 293T cells were transfected with expression plasmids pEF-ELL2-myc, pEF-Tax-1 (1 µg each), both plasmids or the empty vector pEF-1α (mock). After 24 h, cells were stained with primary mouse anti-Tax and rabbit anti-ELL2 antibodies, followed by anti-mouse Alexa Fluor 647 and anti-rabbit Alexa Fluor 555 antibodies, respectively. Staining of nuclei was performed using Prolong Gold reagent with DAPI (4,6-diamidino-2-phenylindole). Images were generated using confocal laser scanning microscope Leica TCS SP5. Images of ELL2 (red), Tax-1 (green), the nucleus (blue), and the merge of all three stains are shown. Scale bars indicate 10 µm. Yellow dots indicate partial co-localizations of Tax-1 and ELL2. (**p**) A region of interest (ROI) is highlighted and (**q**) the graph shows the fluorescence intensities of Tax-1- and ELL2-specific fluorescence along the ROI.

Since our previous work using subcellular fractionation found that both Tax-1 and ELL2 are predominantly localized to the nucleus and identified Tax-1:ELL2 complexes [33], we now sought to define the subcellular localization of ELL2 and Tax-1 in more detail. Therefore, confocal laser scanning microscopy of transiently transfected 293T cells was performed (Figure 1). Upon transfection of 293T cells with EF1a (elongation factor 1α)-driven Tax-1, ELL2, both Tax-1 and ELL2 expression plasmids, or empty vector control (mock) cells were stained at 24 h post transfection with anti-Tax, anti-ELL2, and the respective fluorescently labeled secondary antibodies. Imaging analysis revealed that ELL2 localized in an evenly distributed manner in the nucleus, excluding the nucleoli (Figure 1e,h; red). Unlike ELL2, Tax-1 is found in both the cytoplasm and the nucleus (Figure 1j,l,n,p; green) due to its nuclear–cytoplasmatic shuttling capacity [34,35]. Within the nucleus, Tax-1 accumulates in punctate structures (Figure 1j,l,n,p), which were defined as discrete transcriptional hot spots, so-called Tax-1 nuclear bodies, comprising splicing factors and components of the transcription machinery [35,36]. Staining of Tax-1 and ELL2 revealed that both proteins either localize in close spatial proximity, or Tax-1 and ELL2 colocalize in nuclear bodies (Figure 1p; yellow). Overall, in 69% of all cells expressing both Tax and ELL2 in the nucleus, the co-localization of Tax and ELL2 in nuclear speckles was observable; however, ELL2 expression was also still detectable in nuclear areas surrounding the speckles. The co-localization of Tax-1 and ELL2 was further confirmed by assessing the fluorescence intensities of both Tax-1-and ELL2-specific fluorescence, which exhibited a parallel distribution along a defined region of interest (ROI; Figure 1p,q) in nuclear bodies. These data suggest that ELL2 can either associate or be part of Tax-1 nuclear bodies and support our earlier work showing the co-precipitation of Tax-1 and ELL2 [33].

### 2.2. Point Mutants Impairing Post Translational Modifications of Tax-1 Do Not Affect Tax-1:ELL2 Complex Formation

**Figure 2:**
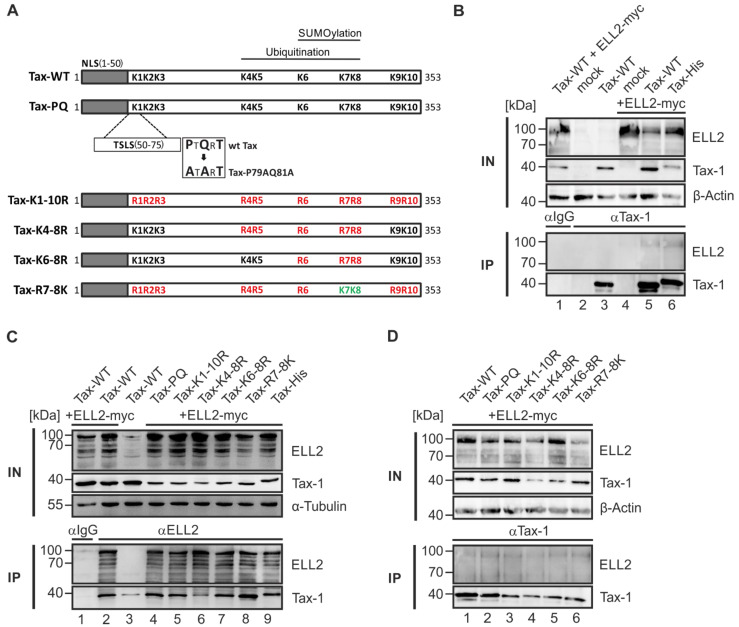
**Tax-1 and ELL2 co-precipitate independent of established Tax-1 point mutations affecting Tax-1 post translational modifications.** (**A**) Schematic overview of Tax-WT (wildtype) and Tax-1 point mutants. The N-terminal nuclear localization signal (NLS, amino acids (aa) 1–50), the Tax-1 speckled structure localization signal (TSLS, aa 50–75) and the potential TRAF-binding motif (PTQRT) and its substitution to an ATART motif in the construct Tax-PQ (Tax P79A Q81A) are indicated. Lysine (K) residues K1-K10 are depicted in black, mutations to arginines (R) in red, and mutations from R to K (based on K1-10R) in green. Lysine residues being critical for ubiquitination and SUMOylation are marked on top. (**B**) 293T cells were transfected with expression plasmids pSG-Tax-WT (Tax-WT), pEF-ELL2-myc (ELL2-myc), pSG-Tax-His (Tax-His; 1 µg each) or the respective empty control vectors pSG5M and pEF (mock; 1 µg each). Each sample was supplemented with the respective empty control vector to a total amount of 2 µg of plasmid DNA if necessary. After 48 h, cells were lysed and 10% of the lysates were taken as input (IN) control. Co-immunoprecipitation (IP) was performed using anti-Tax antibodies or IgG control antibodies. One representative immunoblot out of five independent experiments is shown using antibodies targeting ELL2, Tax-1, β-Actin, and α-Tubulin. (**C**,**D**) 293T cells were transfected with expression plasmids pSG-Tax-WT, pSG-Tax-PQ (Tax-PQ), pSG-Tax-K1-10R (Tax-K1-10R), pSG-Tax-K4-8R (Tax-K4-8R), pSG-Tax-K6-8R (Tax-K6-8R), pSG-Tax-R7-8K (Tax-R7-8K), pSG-Tax-His (Tax-His), and pEF-ELL2-myc (ELL2-myc; 1 µg each). IPs were performed using (**C**) anti-ELL2 or IgG control antibodies or (**D**) anti-Tax antibodies. One representative out of (**C**) four or (**D**) five independent experiments is shown.

**Figure A1:**
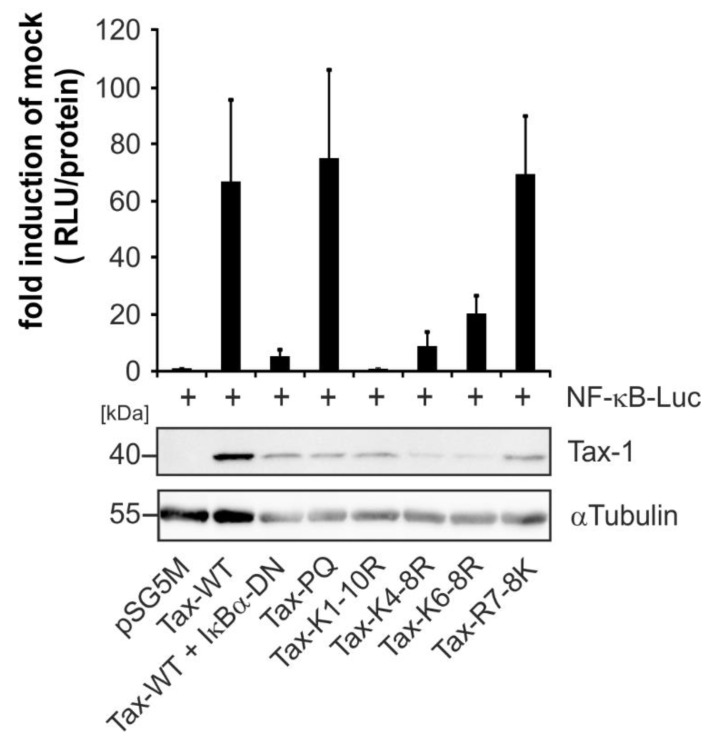
**Tax point mutants differentially activate NF-κB signaling.** The NF-κB reporter plasmid (NF-κB-Luc; 0.1 μg) was transfected together with Tax-1 wildtype (pSG-Tax-WT) or point mutants of Tax-1 (pSG-Tax-PQ, pSG-Tax-K1-10R, pSG-Tax-K4-8R, pSG-Tax-K6-8R, and pSG-TaxR7-8K; 0.01 μg each) in 293T cells. Co-transfection of the dominant-negative mutant IκBα (pIκBα-DN; 0.1 μg) and empty vector (pSG5M) served as negative controls. All transfection mixtures were replenished with pSG5M to 1 μg of total DNA. After 48 h, cells were lysed and analyzed via immunoblot (upper panel) and luciferase assay (lower panel). Relative light units (RLUs) were normalized on protein concentration and on cells co-transfected with pSG5M (mock). The mean values ± SE of four independent experiments, each performed in triplicate, are shown.

The viral protein Tax-1 contains 10 lysine residues at positions 85, 88, 111, 189, 197, 263, 280, 284, 324, and 346 (abbreviated as K1–K10). Earlier work has shown that Tax-1 is post translationally modified by ubiquitination and SUMOylation at lysine residues K4–K8 and K6–K8, respectively [37,38]. Interestingly, post translational modifications (PTMs) of Tax-1 are important for shuttling of Tax-1 between the nucleus and the cytoplasm [34,35]. We sought to test whether Tax-1 PTM mutants that have been shown to be impaired in either ubiquitination, SUMOylation, or both [37,38] also differ in their ability to form complexes with ELL2 (Figure 2A). In the SUMOylation-deficient mutant Tax-PQ (Figure 2A), the potential TRAF-binding motif (PTQRT) was substituted to an ATART motif (Tax P79A Q81A; [37]). In the Tax-K1-10R construct ([37]; Figure 2A), all ten lysine residues were replaced by arginines. In the point mutants Tax-K4-8R and Tax-K6-8R, only lysines four to eight or six to eight, respectively, were replaced by arginines. For the generation of the construct Tax-R7-8K, the mutant Tax-K1-10R was used, and the arginines at position seven and eight were reverted to the wildtype lysine (Figure 2A; [38]). The identity of mutants was not only confirmed by automated sequencing, but also by a functional assay since Tax-1 point mutants are differentially impaired in their capability to induce NF-κB signaling. For this purpose, 293T cells were transfected with an NF-κB-dependent reporter vector and the respective simian virus 40 (SV40)-driven expression constructs of the Tax-1 point mutants (Figure 2A and Figure A1). Compared to Tax-1 wildtype (WT), which was a potent inducer of NF-κB activity, the co-expression of plasmids encoding IκBα-DN, a dominant negative competitor of endogenous IκBα [39] led to a significant decrease in NF-κB-activity, thus confirming the validity of the assay [40]. The Tax-1 point mutant Tax-PQ was comparable to Tax-WT in activating NF-κB activity confirming earlier observations [37]. However, Tax-1 point mutants Tax-K1-10R, Tax-K4-8R, and Tax-K6-8R were impaired in activating NF-κB, while the Tax-1 mutant Tax-R7-8K acted as a potent inducer of NF-κB such as Tax-WT, confirming earlier work [37,38]. Western blot analysis revealed that all mutants were expressed, albeit at varying expression levels (Figure A1). Having shown that Tax-1 point mutants behaved as expected, we performed co-immunoprecipitations between ELL2 and the Tax-1 point mutants to analyze whether Tax-1 PTMs are critical for Tax-1:ELL2 complex formation. For this purpose, 293T cells were transfected with the respective Tax-1 point mutants together with ELL2. First, we controlled Tax-1:ELL2 complex formation by comparing the interaction of ELL2 with Tax-1 wildtype (Tax-WT) or a His-tagged version of Tax-1 (Tax-His) by co-immunoprecipitation (coIP). Compared to precipitations using control antibodies (IgG) in cells expressing both ELL2 and Tax-1 (Figure 2B, lane 1), to mock-transfected cells (Figure 2B, lane 2), or to either Tax-1- or ELL2-expressing cells (Figure 2B, lanes 3 or 4, respectively), ELL2 could be specifically co-precipitated upon the precipitation of Tax-1 in cells that co-expressed ELL2 and Tax-WT or Tax-His (Figure 2B, lanes 5–6). Thus, C-terminal tagging of Tax-1 does not impair Tax-1:ELL2 complex formation. To test the ability of ELL2 to co-precipitate with the Tax-1 PTM constructs, we performed co-IP using anti-ELL2 or IgG control (Figure 2C), or anti-Tax (Figure 2D) precipitation antibodies. Independent of the precipitation antibodies used, all Tax-1 mutants Tax-PQ, Tax-K1-10R, Tax-K4-8R, Tax-K6-8R, and Tax-R7-8K could form complexes with ELL2 comparable to Tax-WT (Figure 2C,D) and to Tax-His (Figure 2C). Occasionally, we found weak co-precipitation of Tax-1 upon observing slight expression levels of endogenous ELL2 in the input fraction (Figure 2C, lane 3), but we could not detect endogenous ELL2 upon precipitation, potentially due to its low stability [26]. Together, our data suggest that Tax-1 PTM affecting ubiquitination and SUMOylation are irrelevant for Tax-1:ELL2 complex formation.

### 2.3. N- and C-Terminal Domains of Tax-1 Are Critical for Tax-1:ELL2 Complex Formation

**Figure 3:**
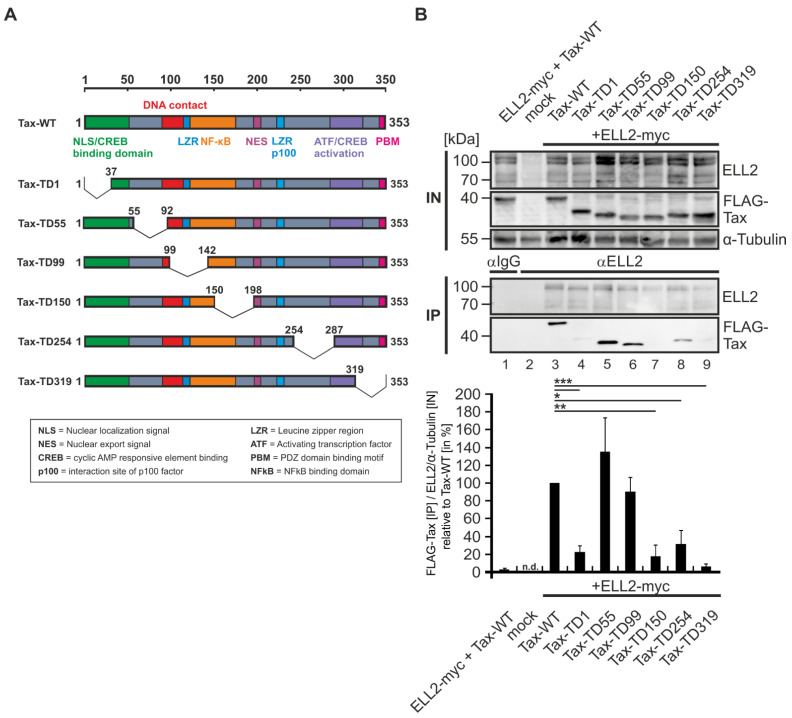
**N-terminal (aa 1–37) and C-terminal (aa 150–353) domains of Tax-1 are critical for Tax-1:ELL2 complex formation.** (**A**) Schematic representation of Tax-1-wildtype (WT) and six truncation mutants harboring deletions beginning from the N-terminus (TD1) to the C-terminus (TD319) of the Tax sequence. Numbers indicate amino acids of Tax-1. (**B**) 293T cells were transfected with 1 µg of the FLAG-tagged expression plasmids pCAG-FLAG-Tax-WT (Tax-WT), Tax truncations pCAG-FLAG-Tax-TD1 (Tax-TD1), pCAG-FLAG-Tax-TD55 (Tax-TD55), pCAG-FLAG-Tax-TD99 (Tax-TD99), pCAG-FLAG-Tax-TD150 (Tax-TD150), pCAG-FLAG-Tax-TD254 (Tax-TD254), and pCAG-FLAG-Tax-TD319 (Tax-TD319) together with pEF-ELL2-myc (ELL2-myc; 1 µg) or the respective empty control vectors pCAG-FLAG and pEF (1 µg each). After 48 h, cells were lysed, and 10% of the lysates were taken as input (IN) control. Co-immunoprecipitations (IPs) were performed using anti-ELL2 or IgG control antibodies. Representative immunoblots out of four independent experiments are shown using antibodies targeting ELL2, FLAG (for detection of Tax), and α-Tubulin. Densitometry was performed to quantitate the amount of co-precipitated FLAG-Tax (Tax) after precipitation of ELL2, and values were normalized on the respective ELL2 expression in the input (normalized on α-Tubulin). Binding of Tax-WT to ELL2 was set to 100%. Bars indicate the means of three independent experiments ± SE and values were compared to Tax-WT using Student’s *t*-test. * *p* < 0.05; ** *p* < 0.01; *** *p* < 0.001; n.d., not determined.

Next, we sought to identify critical domains in Tax-1 that are crucial for Tax-1:ELL2 complex formation. For this purpose, we made use of a panel of FLAG-tagged Tax-1 mutants containing deletions varying from 30 to 50 amino acids [41,42]. Tax-1 WT harbors an N-terminal nuclear localization sequence (NLS), several activation and interaction domains as well as a C-terminal postsynaptic density protein (PSD95), Drosophila disc large tumor suppressor (DlgA), and zonula occludens-1 protein (ZO-1) (PDZ) domain binding motif (PBM), which are sequentially deleted in the depicted mutants (Figure 3A). To explore the potential of these Tax-1 deletions mutants to interact with ELL2, we co-expressed ELL2 with the respective deletion mutants in 293T cells, followed by immunoprecipitations using anti-ELL2 precipitation antibodies. As expected, neither Tax-1 nor ELL2 were precipitated in the isotype control (Figure 3B, lane 1). Interestingly, we could not detect endogenous ELL2 upon ELL2 precipitation (Figure 3B, lane 2), confirming results from Figure 2C (lane 3) and underlining that ELL2 is a highly unstable protein [26]; however, precipitated ELL2 could be detected upon overexpression (Figure 3B, lanes 3–9). While Tax-WT, Tax-TD55, and Tax-TD99 (Figure 3B, lanes 3, 5, and 6) were co-precipitated by ELL2 at comparable levels, the N-terminal deletion mutant Tax-TD1 and the central and C-terminal deletion mutants Tax-TD150, Tax-TD254, and Tax-TD319 (Figure 3B, lanes 4, 7–9) were significantly impaired in interacting with ELL2, which was confirmed by densitometry (Figure 3B, lower part). Thus, both the N-terminus of Tax-1 (aa 1–37) harboring the NLS/CREB binding domain as well as large parts of the central domains and the C-terminus of Tax-1 (aa 150–353) are necessary for Tax-1:ELL2 complex formation.

### 2.4. Tax-2B from HTLV-2B Does Not Interact with ELL2

**Figure 4:**
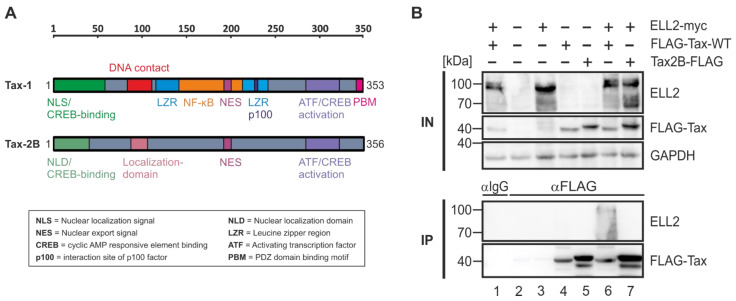
**ELL2 interacts with Tax-1, but not with Tax-2B.** (**A**) Schematic comparison of structural and functional domains of HTLV-1 Tax-1 and HTLV-2 Tax-2B. Numbers indicate amino acids of Tax. (**B**) 293T cells were transfected with expression plasmids pEF-ELL2-myc (ELL2-myc), pCAG-FLAG-Tax (FLAG-Tax-WT), and Tax-2B-FLAG (1 µg each) and supplemented with the respective empty control vectors pEF or pCAG-FLAG to a total amount of 2 μg of DNA, if necessary. After 48 h, cells were lysed, and 5% of the lysates were taken as input (IN) control. The co-immunoprecipitation (IP) was performed using anti-FLAG or IgG control antibodies. One representative immunoblot out of four independent experiments is shown using antibodies targeting ELL2, FLAG (for detection of Tax-1 and Tax-2), and GAPDH.

Since C-terminal deletion mutants of Tax-1 including Tax-TD319 (Figure 3) were not able to interact with ELL2, we had a closer look at the related but non-oncogenic Tax-2B from HTLV-2B, which lacks the C-terminal PDZ-binding motif (PBM; Figure 4A). Briefly, HTLV-1 and the closely related, but non-oncogenic HTLV-2 share similar genome structures; however, in contrast to HTLV-1, HTLV-2 is much less pathogenic, with only a few cases of hairy cell leukemia or neurological diseases being associated with HTLV-2 [20,43,44,45,46,47,48]. There are two major subtypes, HTLV-2A and HTLV-2B, coding for Tax-2A and Tax-2B, respectively, the latter being better understood and studied here [49,50,51,52]. The amino acid sequence similarity of the two proteins Tax-1 and Tax-2B is about 85%. Both Tax proteins (Figure 4A) possess an N-terminal CREB-binding domain (green), an NLS (Tax-1) or NLD (Tax-2B), a central nuclear export sequence (NES, pink), two leucine zipper-like motif regions (LZRs) at aa positions 116–145 and 213–248 (blue), and a C-terminal ATF/CREB activation domain (purple). The main differences between Tax-1 and Tax-2B are the aforementioned Tax-1 PBM (aa 349–353) lacking in Tax-2B and the Tax-1-specific motif from aa 225–232, which enables Tax-1 to activate next to the canonical also the non-canonical NF-κB pathway by interacting with the protein p100 [48,53,54]. To compare the interaction properties of Tax-1 with ELL2 to those of Tax-2B, we transfected 293T cells with ELL2 and FLAG-tagged Tax-1 and Tax-2B expression constructs and performed co-IPs with FLAG-specific antibodies. While the precipitation of Tax-1 led to the co-precipitation of ELL2, the related Tax-2B protein does not interact with ELL2 (Figure 4B, lanes 6–7), indicating that the complex formation with ELL2 is specific for Tax-1, but not for Tax-2B. Although the impact of other differences between Tax-1 and Tax-2B cannot be fully excluded, our data support the hypothesis that the PBM in Tax-1, which is also crucial for T-cell proliferation and viral persistence [55,56], may confer selectivity to Tax-1 for interacting with ELL2.

### 2.5. ELL2 Is a Highly Post Translationally Modified Protein That Contains Two Globular Domains

**Figure 5:**
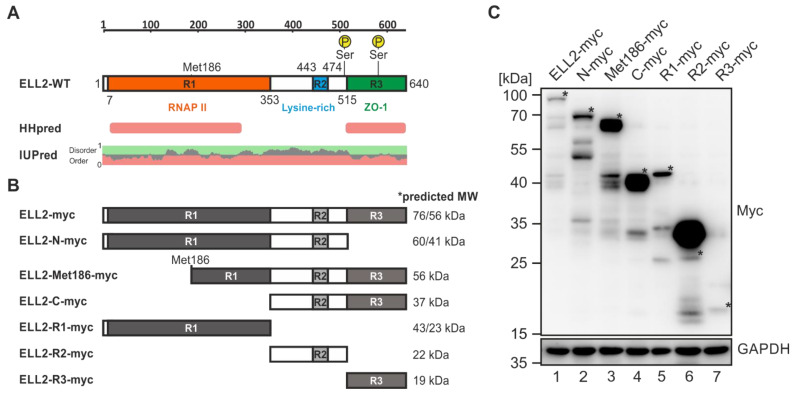
**Characterization of ELL2 truncations and bioinformatic predictions of post translational modifications.** (**A**) Schematic representation of ELL2 wildtype (ELL2-WT) and prediction of globular domains based on bioinformatic predictions using HHpred and IUPred. R1, R2, R3, conserved regions R1, R2, and R3; RNAPII, RNA polymerase II binding; ZO-1, homology to zonula occludens-1; Met186, alternative start codon. P Ser, phosphorylated serine residues. HHpred: Red bars indicate functionally related globular regions predicted through sequence comparisons of homologous, evolutionary related proteins of known 3D structure. IUPred: the prediction profile of protein disorder (green) tendency over the residue position. (**B**) Schematic representation of myc-his-tagged ELL2 truncations together with the respective predicted molecular weight (MW) values for proteins translated from the standard start codon (Met1, first value) and the alternative start codon at Met186 (second value), if applicable. (**C**) Expression analysis of ELL2 truncations. 293T cells were transfected with 1 µg of pEF-1α-driven expression plasmids ELL2-myc, N-myc, Met186-myc, C-myc, R1-myc, R2-myc, and R3-myc. After 48 h, cells were analyzed by Western blot using antibodies specific for Myc and the housekeeping gene GAPDH. * indicates bands corresponding to the predicted sizes (plus post translational modifications) of proteins translated from the first start codon being present.

After narrowing down domains of Tax-1 that are necessary for Tax-1:ELL2 complex formation, we now tried to shed more light on this interaction from the point of view of ELL2. Therefore, we generated ELL2 truncations, focusing on the three regions (Rs), R1, R2, and R3, which are conserved among ELL family members ELL and ELL2 [27]. Briefly, ELL2 wildtype (ELL2-WT; Figure 5A) is composed of an N-terminal R1 (aa 7–353) containing an ELL2 elongation activation domain and RNA Pol II binding domain, a central R2 (aa 443–474) being rich in lysines, and the C-terminal R3 (aa 515–640) binding to the scaffold protein AFF4 within the super elongation complex during HIV proviral transcription [57]. Further, R3 shares structural similarity to the zonula occludens (ZO-1) binding domain of the tight junction protein occludin [27]. In R1, an alternative start codon is located at Met186 [30]. Furthermore, R1 and R2 are bridged by a proline-rich, non-conserved region containing several PXXP motifs, which are potential binding sites for SH3 domains [27]. To avoid the mutagenesis-induced destruction of globular domains, which usually take up enzymatic or functional properties, we first checked the position of globular domains in ELL2 using HHpred [58], IUpred [59,60], and ELM [61] web servers. Structure analysis using HHpred, which makes use of sequence comparisons of homologous, evolutionarily related proteins of known 3D structure, revealed that the regions R1 and R3 of ELL2 harbor globular domains at positions aa 194–292 and at aa 515–638, respectively (Figure 5A). This was to a large extent in accordance with the presence of ordered domains in R1 and R3 as predicted by IUpred (Figure 5B, red), while the central R2 domain and its flanking regions (Figure 5A, aa 353–515) were predicted to be structurally disordered (Figure 5A, IUpred, green). Together, the predicted globular domains located in R1 and R3 have already been assigned functional properties, namely interaction with RNA Pol II and AFF4, respectively [26,27,57,62].

Since the computational analyses suggested that cloning of individual conserved regions from ELL2 should not lead to the destruction of globular domains, we next cloned all possible truncations of these three regions from ELL2 to generate EF1a-driven expression constructs ELL2-N-myc (aa 1–515; R1 and R2), ELL2-Met186-myc (aa 186–640; parts of R1, R2, and R3), ELL2-C-myc (aa 353–640; R2 and R3), ELL2-R1-myc (aa 1–353), ELL2-R2-myc (aa 353–515), and ELL2-R3-myc (aa 515–640; Figure 5B). A schematic overview and the predicted size of the respective mutants is shown in Figure 5B, which took into account the size of the C-terminal myc-his tag, the first start codon (first value), and the alternative start codon at Met186 (second value). After automated sequencing analysis, the newly generated repertoire of ELL2 mutants was further characterized upon transfection of equal amounts of DNA into 293T cells (Figure 5C). Western blot analysis using myc-specific antibodies revealed that all ELL2 mutants were expressed, albeit at varying degrees. Interestingly, the detection of all ELL2 constructs except ELL2-R3-myc (Figure 5C, lane 7) revealed more than one specific band in Western blot analysis. The myc-his-tagged ELL2-WT (Figure 5C, lane 1) does not only express the full length protein, which is heavily post translationally modified as indicated by the higher molecular weight displayed on the Western blot compared to the predicted size (>76 kDa, labeled with *), but it also expresses lower migrating protein species (two bands at ca. 65–70 kDa), which may arise from post translationally modified proteins translated from the alternative start codon Met186 [30]. Additional lower migrating protein species (ca. 40 kDa, three bands) may reflect degradation products of ELL2, which also seem to be post translationally modified. All ELL2 constructs containing R2 (except ELL2-R1-myc and ELL2-R3-myc) exhibited bands with a higher molecular weight than the predicted size (Figure 5C, lanes 1–4 and 6, labeled with *), suggesting that the lysine-rich R2 and its flanking proline-rich region may be responsible for the majority of ELL2’s post translational modifications, which is also supported by the high intensity of the myc-specific band upon expression of ELL2-R2-myc alone (Figure 5C, lane 6). ELL2-R3-myc displays very low expression (Figure 5C, lane 7), which may be due to the high sensitivity of this region towards ubiquitin-dependent degradation (Liu et al., 2012; Yu et al., 2018). Together, the expression of all ELL2 truncations could be confirmed, although at varying expression levels.

### 2.6. N- and C-Terminal Domains of ELL2 Are Critical for Tax-1:ELL2 Complex Formation

**Figure 6:**
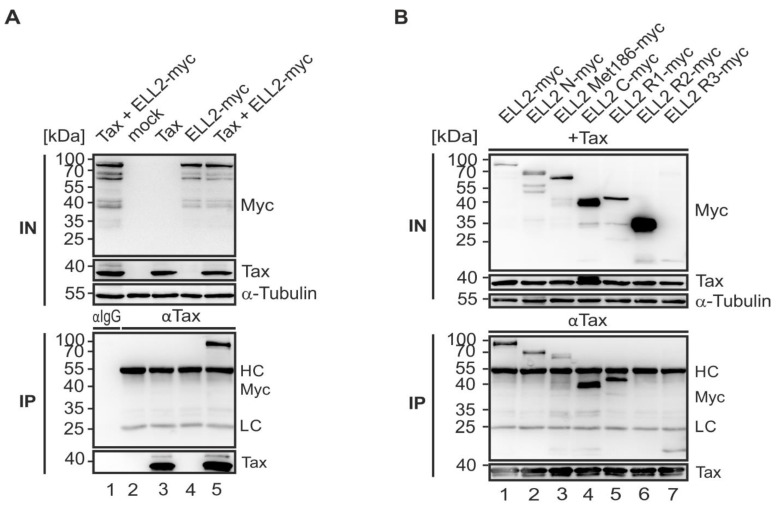

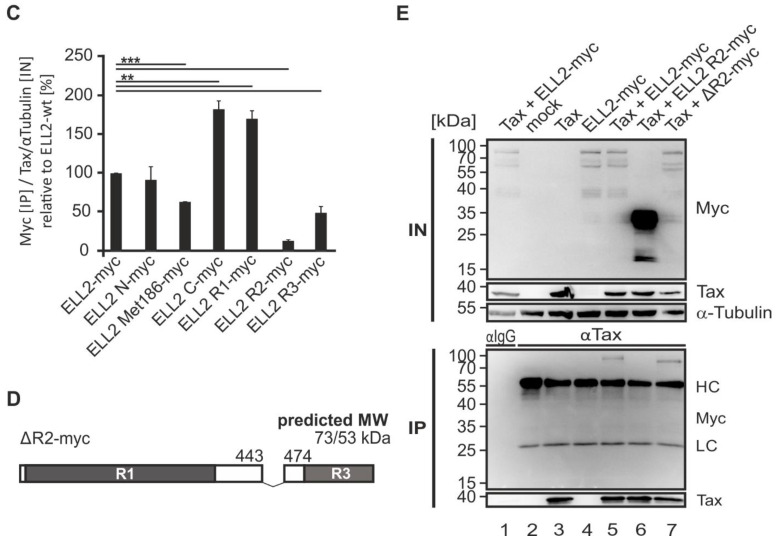
**Multiple domains in ELL2 except ELL2-R2 are important for Tax-1:ELL2 complex formation.** Co-immunoprecipitations (IP) of (**A**) Tax-1 and ELL2 and (**B**) Tax-1 and ELL2 truncations using Tax-specific precipitation antibodies. 293T cells were transfected with expression plasmids pEF-1α-Tax-1 (Tax), pEF-1α-ELL2-myc (ELL2-myc) and ELL2-truncations together with the respective empty control vector. After 48 h, cells were lysed and 10% of the lysates were taken as input (IN) control. IP was performed using anti-Tax antibodies. (**A**,**B**) One representative immunoblot out of five independent experiments is shown using antibodies targeting ELL2, Tax-1, and α-Tubulin. (**C**) Densitometric analysis of three independent experiments +/− SE of co-precipitated ELL2 truncations relative to Tax-1 of the input, normalized on ELL2-WT. Mean values were compared using two-tailed Student’s *t*-test (** indicates *p* < 0.01; *** *p* < 0.001) (**D**) Schematic representation of ΔR2-myc together with the respective predicted molecular weight (MW) values for proteins translated from the standard start codon (Met1, first value) and the alternative start codon at Met186 (second value). (**E**) Co-immunoprecipitation of Tax-1, ELL2, and ΔR2 using Tax-specific precipitation antibodies. One representative out of three independent experiments is shown. IgG, isotype control antibody; HC, heavy chain antibodies; LC, light chain antibodies.

After cloning ELL2 truncations, we next characterized the interaction properties of these constructs with Tax-1. For this purpose, co-immunoprecipitations were performed upon the transfection of 293T cells with EF-1a-driven Tax-1 and ELL2 wildtype expression plasmids. Precipitation with Tax-1-specific antibodies revealed that Tax-1 and ELL2 specifically co-precipitated not only upon expression of SV40- (Figure 2B), but also of EF-1a-driven Tax-1 expression plasmids (Figure 6A, lane 5) confirming earlier findings [33]. Thereafter, we analyzed the panel of newly generated ELL2 mutants and co-expressed them with pEF1a-Tax-1 in 293T cells followed by co-immunoprecipitations using Tax-specific antibodies. The analysis of these newly generated ELL2 mutants revealed that not only ELL2-WT but also all ELL2 mutants co-precipitated with Tax-1, except ELL2-R2, containing the central lysine-rich conserved region R2 (aa 443–474) and a flanking non-conserved proline-rich region, which was impaired in interacting with Tax-1 (Figure 6B, lane 6). Although the ELL2-R2 construct was among the most expressed truncations within the input control, no complex formation with Tax-1 could be detected. However, ELL2-C (aa 354–640), a truncation expressing both R2 and R3, co-precipitated with Tax-1. The expression of C-terminal ELL2-R3 (aa 515–640) was significantly weaker when compared to the other constructs, even though an enrichment of ELL2-R3 could be observed in the IP blot, indicating Tax-1:ELL2 complex formation. ELL2-N and the ELL2-Met186 can both be co-precipitated by Tax-1 at comparable amounts, and ELL2-R1 (aa 1–353), which also binds to RNA polymerase II, is sufficient to mediate interactions with Tax-1 (Figure 6B, lane 5). Densitometric comparison of the binding of ELL2 mutants to Tax-1 with ELL2-WT confirmed these findings and showed that ELL2-R2 was significantly impaired in being co-precipitated by Tax-1 compared to ELL2 -WT (Figure 6C). To analyze this perception in more detail, we generated a new version of ELL2, ΔR2, by deleting the region R2 (Figure 6D) to study whether a missing R2 region interferes with Tax-1:ELL2 complex formation. For this purpose, we performed similar precipitation experiments as conducted for the other ELL2 truncations and compared ELL2-WT to ELL2-R2 and the new construct ΔR2 (Figure 6E). As shown before, Tax-1:ELL2 complex formation was observable for ELL2-WT, but not for ELL2-R2. Interestingly, the new construct ΔR2 was able to be co-precipitated by Tax-1 in comparable amounts to ELL2-WT. In summary, we found that both the C- and N-terminal parts of ELL2 are necessary for Tax-1:ELL2 complex formation while the more centrally localized region R2 is irrelevant for this interaction.

### 2.7. An N-Terminal Conserved Region of ELL2, ELL2-R1, Is Crucial for Enhancing Tax-1-Mediated Transactivation of the HTLV-1 Promoter

**Figure 7:**
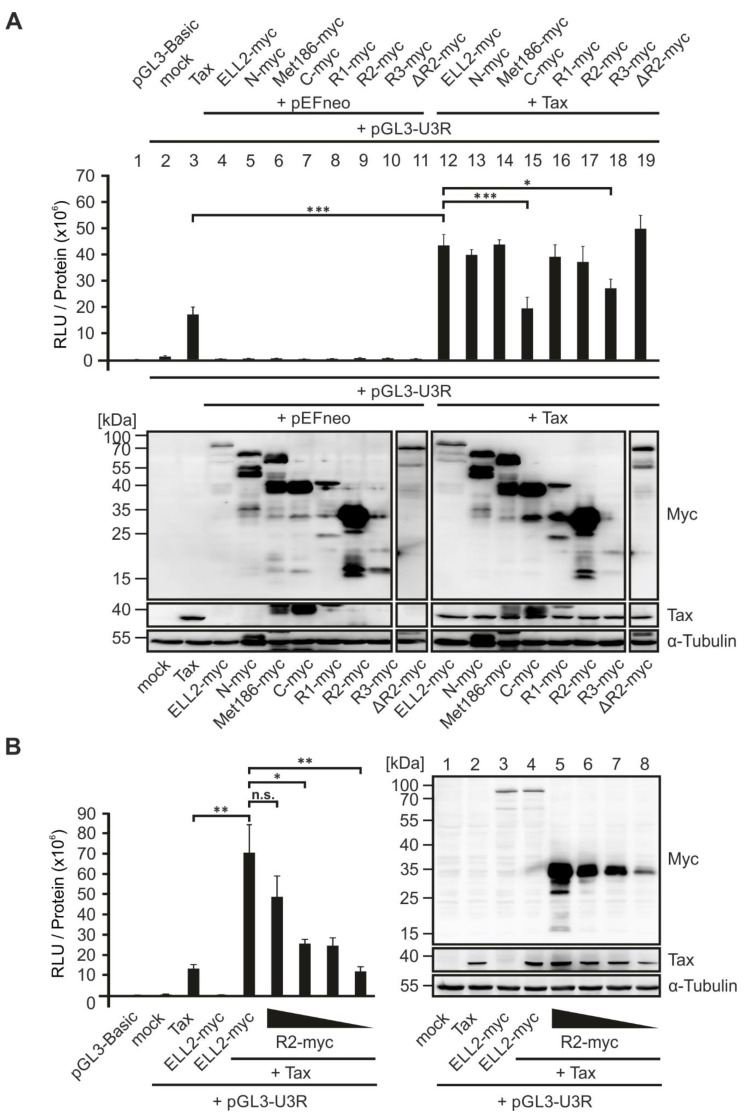
**ELL2-R1 is sufficient for enhancing Tax-1-mediated transactivation of the HTLV-1 promoter.** (**A**) Luciferase reporter gene assays (upper part) and corresponding Western blots (lower part). 293T cells were transfected with expression constructs pEFneo-Tax-1 (Tax; 10 ng), pEF1α-ELL2-myc wt (ELL2-myc) or truncations (890 ng) together with the luciferase reporter construct pGL3-U3R of the HTLV-1 promotor (100 ng each) as indicated. Cells transfected with pGL3-Basic together with Tax and ELL2-myc served as negative control. At 48 h post transfection, luciferase activities were measured in triplicates and relative light units (RLUs) were normalized on protein content of the respective sample. Mean values ±SE are shown and were analyzed by Shapiro–Wilk test, and data pairs were compared using two-tailed Student’s *t*-test (*n* = 3; * *p* < 0.05; *** *p* < 0.001). Western blot to detect expression of ELL2 truncations (anti-myc), Tax-1 and α-Tubulin. Blots were cut due to technical resasons. (**B**) Luciferase reporter assays and Western blots were performed as described in (**A**). 293T cells were transfected with expression constructs pEFneo-Tax-1 (Tax; 10 ng), pEF1α-ELL2-myc wt (ELL2-myc; 890 ng) or decreasing amounts of R2-myc (890 ng, 400 ng, 200 ng, and 100 ng) together with the luciferase reporter construct pGL3-U3R of the HTLV-1 promotor (100 ng each). Mean values ± SE are shown and were analyzed by Shapiro–Wilk test, and data pairs were compared using two-tailed Student’s *t*-test (*n* = 4; * *p* < 0.05; ** *p* < 0.01; n.s., not significant).

Next, we considered the ability of ELL2 truncation mutants to transactivate the viral promotor. Earlier studies have shown that ELL2 enhances Tax-1-mediated activation of the HTLV-1 promoter [33]. Hence, the analysis of the newly generated EF-1a-driven ELL2 constructs could shed light on crucial regions of ELL2 being important for supporting viral transactivation. To this end, we made use of a Tax-1-responsive luciferase-based reporter construct controlled by the U3R fragment of the HTLV-1 promoter [33,63] and performed luciferase assays in 293T cells. As expected, EF-1a-driven Tax-1 significantly increased the activity of pGL3-U3R (Figure 7A, bar 3) compared to an empty vector control (mock; Figure 7A, bar 2) or to the promotor-less pGL3-Basic luciferase vector (Figure 7A, bar 1). None of the constructs from the ELL2 mutant panel nor ELL2-WT were able to enhance the basal transactivation of the HTLV-1 promoter (Figure 7A, bars 4–11), confirming earlier findings with ELL2-WT [33]. Upon co-expression with Tax-1, ELL2-WT was able to increase the Tax-1-mediated transactivation of the HTLV-1 promotor significantly (Figure 7A, bar 12). Moreover, most ELL2 truncations still significantly enhanced the Tax-1-mediated transactivation of the HTLV-1 promotor comparable to ELL2 wildtype (Figure 7A, bars 13,14,16,17,19), except two ELL2-truncations, C-myc (expressing conserved regions R2 and R3) and R3-myc, which failed to enhance Tax-1-mediated U3R activity (Figure 7A, bars 15,18, respectively). While C-myc was expressed very well, R3-myc expression was continuously very low, suggesting that R3 is either not required for ELL2 to foster HTLV-1 promotor activity or that R3 expression is too low to impact transactivation. Among those truncations that enhanced Tax-1-mediated viral transactivation, R2-myc was expressed at exceptionally high levels strongly exceeding expression levels of ELL2-WT. Therefore, we performed additional reporter gene assays upon co-transfection of decreasing concentrations of ELL2-R2 (Figure 7B, left panel). While the highest concentration of R2-myc did not differ from ELL2-WT in enhancing Tax-1-mediated transactivation of the HTLV-1 promoter, decreasing amounts of R2 expression led to a significantly impaired transactivation of the U3R compared to ELL2-WT despite very high expression levels of R2, which were still higher than those of ELL2-WT at every concentration tested. Thus, R2, which is also unable to interact with Tax-1 (Figure 6B,C,E) does not seem to contribute to the Tax-1-mediated transactivation of the HTLV-1 promoter. In summary, comparative analysis revealed that R1-myc, which carries an RNA polymerase II binding domain and contributes to the interaction with Tax-1 (Figure 6B,C), is critical for stimulating Tax-1-mediated HTLV-1 transcription by ELL2.

## 3. Discussion

The HTLV-1 transactivator and oncoprotein Tax-1 is a central player in regulating viral gene expression. In this study, we shed more light on the interplay between Tax-1 and the transcription elongation factor ELL2, which is upregulated in HTLV-1-infected cells [33], and its role in viral transcription, which is potentially a promising target for interfering with HTLV-1 replication. Our study identifies domains in Tax-1 and ELL2 that are crucial for Tax:ELL2 complex formation and for ELL2’s capacity to foster Tax-1-mediated viral transactivation.

Tax-1 is a shuttling protein and can be localized to both the nucleus and the cytoplasm [34,35]. In the nucleus, Tax-1 aggregates in transcriptional hot spots, Tax-1-speckled structures, or nuclear bodies [35,36]. Nuclear bodies are dot-like structures that become visible by staining techniques when Tax-1 is overexpressed [35,36]. Previously, they were thought to represent sites of increased transcriptional activity. It has been shown that, in addition to RNA polymerase II, components of the spliceosome and the P-TEFb subunit CDK9 co-localize with Tax-1 in nuclear bodies [21,35,36]. This study finds that ELL2 also partially co-localizes with Tax-1 in nuclear bodies. ELL2 is the stoichiometrically limiting factor of the super elongation complex (SEC), which is crucial for transcription elongation during HIV infection [26,62,64,65], but ELL2 also plays a role in splicing [30,32]. Thus, the partial co-localization of ELL2 with Tax-1 in nuclear bodies shown here not only confirms Tax:ELL2 complex formation [33] but also argues for increased transcriptional activity of Tax upon the co-expression of ELL2, which we could confirm by luciferase assays. Yet, it remains to be determined whether ELL2 influences alternate exon usage events, which have been described to culminate in ATLL [66].

The ubiquitination and SUMOylation of Tax-1 are known to play important roles in the activation of the NF-κB signaling pathway and the subcellular localization of Tax-1 [34,38]. The present study confirms that point mutants of Tax-1, in which lysines important for ubiquitination and SUMOylation or the potential TRAF-binding motif (PTQRT; PxQxT) are mutated, can differentially stimulate NF-κB activity [37,38]. We also found that lysine mutations or mutations of the PxQxT motif in Tax-1 do not affect the ability of Tax-1 to interact with ELL2. Making use of Tax-1 deletion mutants [41], we found that both the N-terminus (up to amino acid 37) and the C-terminus (starting at amino acid 150) of Tax are important for Tax-1:ELL2 complex formation. Since more than one deletion mutant of Tax-1 was impaired to interact with ELL2, it is conceivable that Tax-1:ELL2 complex formation does not necessarily rely on a direct interaction, but on indirect interactions in the context of a multi-component protein complex. The TD1 mutant (Δ1-37) lacks parts of the nuclear localization sequence (aa 1–55) and the zinc finger motif (aa 22–55) [67,68]. In addition, the region comprising amino acids 1 to 55 is important for CREB activation [69]. Known binding partners for this region include CREB/ATF, TATA binding protein (TBP), p62, and cyclin D/cyclin dependent kinase (CDK) [70]. Starting at amino acid 150, deletions in Tax-1 also appear to result in decreased interaction with ELL2, particularly in the regions from 150 to 198, 254 to 287, and 319 to 353, where large portions of the nuclear export signal (aa 188–200) are located [71]. The region from 113 to 258 is also important for NF-κB activation [67,72], and amino acids 127 to 228 play a role in Tax-1 dimerization [73]. The C-terminal PDZ binding motif (aa 350–353) is crucial for interaction with various proteins, such as human disc large (hDlg) and human homolog Scrib (hScrib) [74,75]. At amino acid 346, Tax-1 interacts with p300 and is thereby acetylated, which presumably occurs in nuclear bodies and is required for NF-κB activation [76,77]. Interestingly, amino acids 289 to 353 are important not only for the interaction of Tax-1 with ELL2, but also for the interaction of Tax-1 with the P-TEFb subunit cyclin T1 [21,78]. Since it is known that ELL2 is recruited by the HIV transactivator protein Tat into a protein complex called super elongation complex (SEC) together with P-TEFb and directly interacts with the scaffold protein AFF4 [26,62], it would now be interesting to see in future studies that address whether Tax-1 and ELL2, together with P-TEFb and the scaffold protein AFF4, are also part of a common complex similar to the super elongation complex described for HIV Tat. This idea is supported by our finding that the C-terminal region of ELL2, which binds AFF4 in the HIV super elongation complex [26,62], is also important for Tax-1:ELL2 complex formation. Moreover, ELL2 R1, which is crucial for transcription elongation [27], is also able to complex with Tax-1.

In this work, we report that the non-oncogenic Tax-2B from HTLV-2 does not interact with ELL2. Briefly, compared to HTLV-1, HTLV-2 is not considered to cause ATL, potentially due to differences in the viral Tax-1 and Tax-2 and HBZ (HTLV-1) and APH2 (HTLV-2) proteins, respectively [48]. An important difference between Tax-1 and Tax-2B is the absence of the C-terminal PDZ binding motif (PBM) in Tax-2B. The PBM in Tax-1 is not only important for a variety of protein–protein interactions, but also for the Tax-mediated promotion of T-cell proliferation, viral transformation, and viral persistence [54,55,56,79,80] The particular importance of the PBM for Tax-1:ELL2 complex formation is further supported by our findings that Tax-1 deletion mutant TD319 lacking the PBM is not co-precipitated by ELL2. Further, interactions of Tax-2B with P-TEFb, which could be important for an interaction with ELL2, have not been described so far. Together, the specificity of ELL2 to interact with Tax-1 but not with Tax-2 suggests that ELL2 could contribute to Tax-1’s oncogenic potential.

ELL2 harbors an alternative start codon at position 186 resulting in a fragment with a molecular weight of ca. 58 kDa. Another fragment with the size of 59 kDa is most presumably generated by several tryptic cleavage sites near the the alternative start codon [30]. Furthermore, an isoform of ELL2 with an estimated size of 45 kDa was described [81] lacking amino acids 140–389. Moreover, ELL2 contains an occludin-like domain in its C-terminal R3 region, which has been structurally resolved in complex with an AFF-peptide. The ELL2 C-terminus has been described as “ELLbow”, an arch-shaped domain similar to the cellular protein occludin [57]. Further, ELL2 is heavily post translationally modified. In this work, the newly cloned ELL2 deletion mutants allowed us to gain some insights into post translational modifications of ELL2. ELL2 WT and ELL2 mutants containing R2 showed a characteristic band pattern in immunoblot: (1) one band reflecting a higher molecular weight variant of ELL2, suggesting the presence of PTMs; (2) one or two bands that could arise either from an alternative start codon at Met186 and PTMs thereof, or from degradation products of ELL2. ELL2 contains some conserved AUGs (methionine) at which protein synthesis can alternatively start, which might explain the different variants of ELL2. Moreover, potential arginine–lysine–trypsin cleavage sites have been described in ELL2 around Met186, which can be cut by stress-induced proteases and thus could also be responsible for truncated degradation products of ELL2 [30]. The banding pattern additionally suggests different variants of ELL2, for example, due to different post translational modification. ELL2 is polyubiquitinated by the E3 ubiquitin ligase Siah1. Ubiquitination usually occurs at lysines, which are found in increased numbers in the lysine-rich region R2 of the protein [27,82]. In addition, ELL2 is thought to be phosphorylated at two serines (S503, S580; Figure 5A) [83,84,85]. In contrast to ELL2-WT and the other ELL2 truncations, only a single band was detected when ELL2-R3 was expressed, suggesting that N-terminal regions are subject to stronger modifications than C-terminal regions of ELL2. Since phosphorylation sites in ELL2 have only been determined by high-throughput mass spectrometry so far, experimental verification of these and other PTMs is still lacking.

ELL2 is a highly unstable protein with a short half-life and is targeted by the E3 ubiquitin ligase Siah-1 for ubiquitination and proteasomal degradation. Specifically, the C-terminal region of ELL2 (aa 532–640) confers high sensitivity to Siah-1-induced degradation, although it does not appear to contribute prominently to ELL2:Siah1 interaction [82,86]. In uninfected primary T cells or T-cell lines, ELL2 protein expression is not detectable [33], suggesting that ELL2 protein is either continuously degraded, or, depending on the cell line studied, that *ELL2* transcripts are already impaired [33]. Since ELL2 is highly expressed in HTLV-1-infected cells [33], our data showing that Tax-1:ELL2 complex formation also occurs via the C-terminus of ELL2 suggests that Tax:ELL2 complex formation could prevent ELL2 from degradation in HTLV-1-transformed cells. However, additional work is needed to clarify this issue.

In addition to the interaction analysis of ELL2 with Tax-1, ELL2 truncation mutants were screened for their ability to transactivate the HTLV-1 promoter together with Tax-1. Our study extends earlier findings by Mann et al. [33] showing that besides ELL2 WT, all ELL2 truncations harboring R1 achieved a significant increase in HTLV-1 promoter activity when co-expressed with Tax-1. In contrast to ELL2-WT, the ELL2 truncations C-myc and R3-myc, both containing R3, could not enhance the Tax-1-mediated activation of the HTLV-1 promoter, suggesting that while being important for ELL2 stability [82,86], R3 seems to be neglectable for the Tax-1-mediated transactivation of the HTLV-1 promoter. Next, our data suggests that R1, which carries an RNA pol II binding domain, is required for Tax-1:ELL2 complex formation and for an increase in promoter activity since all ELL2-truncations carrying R1 were able to enhance Tax-1’s capacity to transactivate the HTLV-1 promoter. However, our analyses show that differences in expression levels of the ELL2 mutants could bias the interpretation of the reporter gene assays, as exemplified with the ELL2 truncation R2-myc. While being able to enhance Tax-1-mediated transactivation of the HTLV-1 promoter comparable to ELL2-WT, this effect was lost when R2-myc expression levels were adjusted to those of ELL2-WT, suggesting that R2 does not impact viral transactivation. Since ELL2 increases not only the Tax-1-mediated regulation of the HTLV-1 promoter but also that of a cellular CREB-dependent promoter [33], studying the interaction between Tax-1 and ELL2 is also relevant for elucidating gene regulation during viral transformation. In summary, this work further characterized the interaction of the viral oncoprotein Tax-1 with the transcription elongation factor ELL2 using different Tax-1 and ELL2 mutants. In contrast to the oncoprotein Tax-1 from HTLV-1, the transactivator Tax-2 from HTLV-2 does not interact with ELL2. Thus, ELL2 may be a cellular mediator of the oncogenic potential of Tax-1/HTLV-1.

## 4. Materials and Methods

### 4.1. Cell Culture

HEK 293T cells were cultured in Dulbecco’s modified Eagle medium (GIBCO, Life Technologies) containing 10% fetal calf serum, L-glutamine (0.35 mg/mL), 0.12 mg/mL penicillin and 0.12 mg/mL streptomycin.

### 4.2. Plasmids and Cloning

The following plasmids were used: pEF-1α and pEFneo (Life Technologies, controls); the ELL2 expression plasmid pEF-1α-ELL2-myc (pEF-1α-ELL2) [33]; the luciferase reporter control vector pGL3-Basic (Promega, Mannheim, Germany); the luciferase reporter vector harboring the U3R sequence of the HTLV-1 LTR pGL3-U3R-Luc (U3R-Luc) [33]; the luciferase reporter vector pGL3-NF-κB-Luc (NF-κB-Luc) containing the luc gene under control of the five NF-κB responsive elements (Stratagene, La Jolla, CA, USA); the expression plasmid for a dominant-negative variant of IκBα, inhibitor of NF-κB, IκBα-DN, cloned into pcDNA [39]; the Tax expression vectors pEFneo-Tax1 (kindly provided by Masahiro Fujii) [53], and pEF-Tax1 [40]; the Tax-1 expression vector FLAG-Tax-WT (pCAG-FLAG-Tax-wt), the respective FLAG-tagged Tax-deletion mutants Tax-TD1, Tax-TD55, Tax-TD99, Tax-TD150, Tax-TD254, Tax-TD319 Tax, and the control vectro pCAG-FLAG were kindly provided by Jean-Marie Peloponese [41,42]; Tax2B-FLAG (Tax-2F in pcDNA6.2/N) was kindly provided by Umberto Bertazzoni [44]; the Tax expression vector Tax-His (pSG-Tax-6-His), Tax-WT (pSG-Tax-WT), and the respective point mutants Tax-PQ (pSG-Tax-PQ), Tax-K1-10R (pSG-Tax-K1-10R), Tax-K4-8R (pSG-Tax-K4-8R), Tax-K6-8R (pSG-Tax-K6-8R), and Tax-R7-8K (pSG-Tax-R7-8K) were kindly provided by Claudine Pique [37,38]. For cloning of all C-terminally myc-tagged ELL2 truncations, pEF1α-ELL2-myc (also containing a C-terminal polyhistidin tag) was used as template. Primers were designed to contain BamHI and NotI restriction sites and Vent DNA Polymerase (New England Biolabs) was used for PCR: denaturation for 40 s at 94 °C, annealing for 40 s at 58 °C, and polymerization for 30–90 s at 72 °C (35 cycles). The ELL2-truncations, their amino acid composition compared to ELL2 wildtype and the respective primers used are listed: pEF-1α-ELL2 N-myc, aa 1–515, ELL2N-fwd: 5′-TGAGGATCCATGGCGGCGGGG-3′, ELL2N-rev: 3′-TGAGCGGCCGCGCAGTGCAAT-CCTC-5′; pEF1α-ELL2 Met186-myc, starting at the alternative startcodon Met186, aa 186–640, ELL2-Met186_Fwd: 5′-TGGGGATCCATGAACCCTGCAAATACAATTCG-3′, ELL2-Cmyc_Rev-640: 3′-AGAGCGGCCGCAAGGACCATGACTCTGC-5′; pEF1α-ELL2 C-myc, aa 353- 640, ELL2-Cmyc_Fwd-354: 5′-TCGGGATCCATGCCCACCAGTG-AAAAATCG-3′, ELL2-Cmyc_Rev-640: 3′-AGAGCGGCCGCAAGGACCATGACTCTGC-5′; pEF1α-ELL2 R1-myc, aa 1–353, ELL2-R1_Fwd-1: 5′-TCGG-GATCCATGGCGG-CGGGGGGGACAGG-3′, ELL2-R1_Rev-353: 3-‘CAGGCGGCC-GCAAATTCAAATGAC-CATTTAGTG-5′; pEF1α-ELL2 R2-myc, aa 353–515 ELL2-Cmyc_Fwd-354: 5′-TCGG-GATCCATGCCCACCAGTGAAAAATCG-3′, ELL2N-rev 3′-TGAGCGGCCGCGCAG-TGCAATCCTC-5′; pEF1α-ELL2 R3-myc, aa 515–640, ELL2-R3-Fwd: 5′-TAGGATCC-ATGACTGCCTCCAT-3′, ELL2-Cmyc_Rev-640: 3′-AGAGCGGCCGCAAGGACCATGA-CTCTGC-5′. The construct pEF1α-ELL2 ΔR2-myc is composed of full-length ELL2 harboring a deletion of region R2 (aa 443–474) and was cloned by a five-step overlap extension PCR. All five PCR steps were performed using Vent Polymerase in 25 cycles (denaturation for 45 s at 94 °C, annealing for 60 s at 58 °C and polymerization for 120 s at 72 °C. For the first PCR, the primers ELL2-R1_Fwd-1 5′-TCGGGATCCATGGCGG-CGGGGGGGACAGG-3′ and ELL2-R1-R3_Rev 5′-GTGCTTTTTTATGGAACCAGGGGG-TAAGGT-3′ were used; for the second PCR, ELL2-R1-R3_Fwd 5′-ATAAAAAAGC-ACGACATTGAGACTATTG-3′ and ELL2-Cmyc_Rev-640 3′-AGAGCGGCCGCAA GGACCATGACTCTGC-5′ were used; for the third PCR, ELL2-R1_Fwd-1 5′-TCGGGATCCATGGCGGCGGGGGGGACAGG-3′ and Delta R2 Rev 5′-GTCGTGCT-TTTTTATGGAACCAGGGGGTAAGG-’3 were used; for the fourth PCR, Delta R2 Fwd 5′-CCTTACCCCCTGGTTCCATAAAAAAGCACGAC-’3 and ELL2-Cmyc_Rev-640 3′-AGAGCGGCCGCAAGGACCATGACTCTGC-5′ were used; and the last PCR was performed with ELL2-R1_Fwd-1 5′-TCGGGATCCATGGCGGCGGGG-GGGACAGG-3′ and ELL2-Cmyc_Rev-640 3′-AGAGCGGCCGCAAGGACCATGACT-CTGC-5′. After each PCR step, the products were purified from agarose gels using a gel extraction kit (Qiagen) and served as templates in the subsequent PCR.

### 4.3. Transient Transfections

For transient expression experiments, 293T cells were seeded at 5 × 10^5^ cells per six-well plate. After 24 h, cells were transfected using GeneJuice transfection reagent (Merck Millipore, Darmstadt, Germany) according to the manufacturer’s protocol using a total amount of 2 μg of DNA. Cells were lysed 48 h after transfection. For luciferase reporter assays, 2.5 × 10^5^ 293T cells were seeded in a 12-well format. At 24 h after seeding, 293T cells were co-transfected with a total amount of 1 µg of plasmid DNA.

### 4.4. Western Blot and Antibodies

Transfected cells were lysed using TNE lysis buffer (10 mM NaCl, 10 mM Tris/HCl (pH 7.0), 10 mM EDTA, 1% Triton X-100, 2 mM DTT and protease inhibitors (20 µg/mL leupeptin, 20 µg/mL aprotinin and 1 mM phenylmethylsulfonyl fluoride)) and subjected to repeated freeze-and-thaw cycles between −196 °C (liquid nitrogen) and 30 °C (thermo block). Afterwards, cells were additionally sonicated three times for 30 s (Branson Ultrasonics Analog Sonifier Modell 450, output control = 8, duty cycle = 60, 3 × 30 s). Protein lysates were centrifuged, and the supernatants were adjusted to equal amounts of proteins (30–60 µg) and boiled for 10 min at 95 °C in sodium dodecyl sulfate (SDS) loading dye (10 mM Tris/HCl (pH 6.8), 10% glycerol, 2% SDS, 0.1% bromophenol blue, 5% β-mercaptoethanol). Protein samples were separated by SDS-PAGE and transferred to nitrocellulose membranes (Whatmann, Protran, Whatmann GmbH, Dassel, Germany) using standard techniques. As molecular weight marker, PageRuler Prestained Protein Ladder (Thermo Scientific) was used. Proteins were detected with the following primary antibodies: mouse anti-Tax-1 (derived from the hybridoma cell line 168B17-46-34, provided by B. Langton through the AIDS Research and Reference Reagent Program, Division of AIDS, NIAID, NIH [87], mouse monoclonal anti-α-Tubulin (T9026, Sigma), mouse monoclonal anti-β-Actin (AC-15, Sigma), mouse monoclonal anti-GAPDH (3B1E9, GenScript), mouse monoclonal anti-Flag (M2, Sigma-Aldrich), rabbit polyclonal anti-ELL2 (A302-505A, Bethyllabs), mouse monoclonal anti-Myc (9B11, Cell Signaling). Horseradish peroxidase-coupled secondary antibodies were obtained from GE Healthcare (GE Healthcare, Little Chalfont, UK). Peroxidase activity was assessed by enhanced chemiluminescence using a CCD camera (ChemoStar, Intas Science Imaging GmbH, Göttingen, Germany). Intensities of specific bands of proteins of interest were quantitated using Advanced Image Data Analyzer (AIDA Version 4.22.034, Raytest Isotopenmessgeräte GmbH, Straubenhardt, Germany).

### 4.5. Luciferase Reporter Assays

For luciferase reporter assays, 2.5 × 10^5^ 293T cells were seeded in a 12-well format in quadruplicates, and 24 h later, cells were transfected with a total amount of 1 µg of DNA as indicated in Section 4.3. After 48 h, three samples per approach were lysed for the luciferase reporter assays, which were performed as described previously [33]. The remaining fourth sample was used for Western blot analysis (see Section 4.4). To determine the activity of the HTLV-1-U3R-Luc reporter, relative light units (RLUs) were normalized on the respective amount of protein as measured via Bradford assay. Background activity was determined by measuring activity of the negative control vector pGL3-Basic.

### 4.6. Co-Immunoprecipitation

For co-immunoprecipitation, 5 × 10^5^ 293T cells were seeded on 6-well plates in duplicate, transfected with a total amount of 2 µg of the indicated plasmids and lysed as described in Section 4.4. A total of 10% of the lysates were taken as input control (IN) and the rest of the lysates were subjected to co-immunoprecipitation (coIP). For the coIP, the remaining 90% of lysates were rotated and pre-incubated with anti-Tax antibodies [87], anti-ELL2-antibodies, anti-FLAG, or isotype control antibodies over night at 4 °C. After 12 h, pre-washed protein G-coupled magnetic beads (Dynabeads; Life Technologies) were added to the lysates followed by rotation for 1 h at 20 °C. After precipitation, the IP samples were washed in TNE lysis buffer and eluted in 50 mM glycine (pH 2.6). Both the IN and the IP samples were boiled in sodium dodecyl sulfate (SDS) loading dye (10 mM Tris/HCl (pH 6.8), 10% glycerol, 2% SDS, 0.1% bromophenole blue, 5% β-mercaptoethanol) at 95 °C for 10 min. All lysates were then subjected to SDS–polyacrylamide gel electrophoresis (SDS-PAGE) and transferred to nitrocellulose membranes using standard techniques. Densitometry using AIDA was performed to quantitate the amount of co-precipitated FLAG-Tax (Tax) after the precipitation of ELL2, and values were normalized on the respective ELL2 expression in the input (normalized on α-Tubulin). Binding of Tax-WT to ELL2 was set to 100%. The binding of co-precipitated ELL2 truncations to Tax after FLAG (Tax)-IP was calculated by dividing the band intensities of ELL2 truncations (IP) by the quotient [Tax(IN)/Tubulin(IN)] and normalized on binding of wildtype ELL2-myc (100%).

### 4.7. Immunofluorescence and Confocal Laser Scanning Microscopy

To investigate the subcellular localization of Tax and ELL2, 1.5 × 10^5^ 293T cells/experiment were seeded on coverslips. After 24 h, cells were transfected with expression plasmids pEF1α-ELL-myc (1 µg) or pEF1alpha Tax (1 µg), supplemented with the respective empty control vector pEF-1α to 2 µg. After another 24 h, cells were fixed with 4% para-formaldehyde (PFA, 45 min, humid chamber, 20 °C), washed twice in PBSo (phosphate-buffered saline without Ca^2+^ and Mg^2+^), and permeabilized with 0.2% Triton X-100 (20 min, 4 °C). After three wash steps, unspecific binding was prevented by the incubation of cells in PBSo with 5% FCS/1% BSA for 1 h at 20 °C in a humid chamber. After this blocking step, cells were washed three times with PBSo/5% FCS/1% BSA. Subsequently, cells were stained with primary antibodies mouse anti-Tax (1:2) [87] and rabbit polyclonal anti-ELL2 (1:200; A302–505A, Bethyllabs) in PBSo/5% FCS/1% BSA (30 min, 37 °C in humid chamber) followed by washing and secondary antibodies anti-mouse AlexaFluor 647 and anti-rabbit AlexaFluor 488 in PBSo/5% FCS/1% BSA (1:200 each, Life Technologies, 1 h, humid chamber, 37 °C). After three final washing steps with PBSo, slides were covered with ProLong Gold antifade reagent with DAPI (Life Technologies) and analyzed by confocal laser scanning microscopy. All images were acquired using a Leica TCS SP5 confocal laser scanning microscope equipped with a 63 × 1.4 HCX PL APO CS oil immersion objective lens. LAS AF software (Leica, Wetzlar, Germany) was used for the analysis of images. To quantitate the frequency of Tax^+^ELL2^+^ cells in which ELL2 forms nuclear speckles with Tax, 120 Tax^+^ELL2^+^ cells in eight different optical fields were evaluated.

### 4.8. Statistics

For statistical analysis, the Shapiro–Wilk test was used as indicated followed by a two-tailed Student’s *t*-test using GraphPad Prism and Microsoft Excel. *p*-values below 0.05, 0.01, or 0.001 were indicated as significant (*), very significant (**), or highly significant (***), respectively.

### 4.9. Bioinformatics

To detect globular protein domains in ELL2, the ELL2 protein sequence (UniProt ID O00472) was analyzed by using HHpred [58], https://toolkit.tuebingen.mpg.de/tools/hhpred; last access on 9 June 2021), IUPred2A [60], https://iupred2a.elte.hu/; last access on 9 June 2021) and the Eukaryotic Linear Motif resource for Functional Sites in Proteins (ELM; http://elm.eu.org/; last access on 16 December 2021) [61]. The molecular weight of myc-his-tagged ELL2 constructs was predicted with the protein molecular weight tool provided from bioinformatics.org.

## Data Availability

All data are provided in the manuscript.
